# Profiling of the Molecular Weight and Structural Isomer Abundance of Macroalgae-Derived Phlorotannins

**DOI:** 10.3390/md13010509

**Published:** 2015-01-16

**Authors:** Natalie Heffernan, Nigel P. Brunton, Richard J. FitzGerald, Thomas J. Smyth

**Affiliations:** 1 Food Biosciences Department, Teagasc Food Research Centre, Ashtown, Dublin 15, Ireland; E-Mail: natalie.heffernan@teagasc.ie; 2 Department of Life Science, University of Limerick, Limerick, Ireland; E-Mail: dick.fitzgerald@ul.ie; 3 School of Agriculture and Food Science, University College Dublin, Belfield, Dublin 4, Ireland; E-Mail: nigel.brunton@ucd.ie; 4 Department of Life Sciences, Institute of Technology Sligo, Sligo, Ireland

**Keywords:** phlorotannins, metabolomic profiling, UPLC-MS/MS profiling, antioxidant activity, isomerization, brown macroalgae

## Abstract

Phlorotannins are a group of complex polymers of phloroglucinol (1,3,5-trihydroxybenzene) unique to macroalgae. These phenolic compounds are integral structural components of the cell wall in brown algae, but also play many secondary ecological roles such as protection from UV radiation and defense against grazing. This study employed Ultra Performance Liquid Chromatography (UPLC) with tandem mass spectrometry to investigate isomeric complexity and observed differences in phlorotannins derived from macroalgae harvested off the Irish coast (*Fucus serratus*, *Fucus vesiculosus*, *Himanthalia elongata* and *Cystoseira nodicaulis*). Antioxidant activity and total phenolic content assays were used as an index for producing phlorotannin fractions, enriched using molecular weight cut-off dialysis with subsequent flash chromatography to profile phlorotannin isomers in these macroalgae. These fractions were profiled using UPLC-MS with multiple reaction monitoring (MRM) and the level of isomerization for specific molecular weight phlorotannins between 3 and 16 monomers were determined. The majority of the low molecular weight (LMW) phlorotannins were found to have a molecular weight range equivalent to 4–12 monomers of phloroglucinol. The level of isomerization within the individual macroalgal species differed, resulting in substantially different numbers of phlorotannin isomers for particular molecular weights. *F. vesiculosus* had the highest number of isomers of 61 at one specific molecular mass, corresponding to 12 phloroglucinol units (PGUs). These results highlight the complex nature of these extracts and emphasize the challenges involved in structural elucidation of these compounds.

## 1. Introduction

The biological activities of marine organisms have been studied intensely and many species have been shown to have potent antioxidant, anti-inflammatory, anti-microbial, and neuroprotective activity both *in vitro* and *in vivo* [1,2,3,4,5]. In particular, brown algae are reported to contain higher levels of polyphenols in comparison to red and green algae [6]. Brown macroalgae are a well-known source of structurally unique polyphenols known as phlorotannins, derived from the oligomerization and decoupling of the monomer phloroglucinol (1,3,5-trihydroxybenzene), with molecular weights ranging from 126 Da to 100 kDa [7,8,9]. Research suggests that these compounds act as a defense mechanism within macroalgae against herbivores [10,11,12,13], microbes [14,15], and the detrimental effects of ultraviolet (UV) radiation [16]. Phlorotannins also have allelopathic activity against epibionts [17], and are important for cell wall development at early phases of zygote growth in the Fucaceae family [18,19]. The relative abundance of phlorotannins (5%–30% of the dry weight of the algae), in particular macroalgal species and their known biological activity, has stimulated considerable research into their potential uses in a range of therapeutics. Reported bioactivities and beneficial health effects of phlorotannins include antioxidant properties, anti-allergic effects, anti-inflammatory activity, anti-HIV-1 activity, anti-carcinogenic activity, anti-diabetic activity, and acting as chemopreventive agents, anti-plasmin, and HAase inhibitors [20,21,22,23,24,25,26,27,28,29,30,31,32,33]. However, relatively limited characterization of macroalgae-derived phlorotannins has been carried out. This is possibly a consequence of the structural complexity that can arise due to the polymeric nature of this group of compounds, resulting in variation in both the number of monomers present and the positions at which they are linked. Predominantly, only low molecular weight phlorotannins of 2–8 monomeric units have been characterized in the species *F. vesiculosus* [30] and *Ecklonia cava* [31]. This represents only a small proportion of the phlorotannins present in *F. vesiculosus*, according to reports from Wang *et al.* [5]. Wang *et al.* [5] reported that polyphenols in *F. vesiculosus* were found to consist mainly of high molecular weight phlorotannin polymers. However, to realize the full potential of algal phlorotannins a deeper understanding of their structural complexity within individual macroalgae species is required. The highly complex nature of phlorotannin composition has meant that relatively few structures have been successfully elucidated or detected. However, the availability of advanced chromatographic and mass spectrometric techniques opens up the possibility of more in-depth studies of the isomeric complexity of phlorotannins. In particular, the use of ultra performance liquid chromatography (UPLC) with triple quadrupole tandem mass spectrometry (UPLC-QQQ-MS) couples the improved resolution power of reduced particle size UPLC columns (<2 μm) with the scanning speeds and sensitivity of a triple quadrupole mass spectrometer. UPLC-MS applications for the analysis of phlorotannins from various species of brown algae (*F. vesiculosus*, *Ascophyllum nodosum*, *Pelvetia canaliculata*) have been previously reported in the literature [32,33]. However, this is the first report observing complex chromatographic separation and metabolomic profiling of low molecular weight phlorotannins consisting of more than 10 units of phloroglucinols from the species in this study. Montero *et al.* [34] reported the separation and characterization of phlorotannins containing from 5 to 17 phloroglucinol units in the brown algae *Cystoseira abies-marina* by HILIC × RP-DAD-MS/MS. Steevensz *et al.* [35] reported the level of isomerization for specific molecular weight phlorotannins between 3 and 16 monomers.

Therefore, the main objective of the present study was to use UPLC with tandem mass spectrometry in multiple reaction monitoring (MRM) mode as a tool to investigate the isomeric complexity of enriched phlorotannin extracts derived from a selection of sustainable macroalgal species harvested off the Irish coast (*F. serratus*, *F. vesiculosus*, *H. elongata* and *C. nodicaulis*). Information of this nature is not only important with regards to describing the isomeric complexity of phlorotannins but also in highlighting the differences between different macroalgal species that may present significant stumbling blocks to their possible structural characterization and use as therapeutic agents. This information also serves to highlight that although research in this area has been carried out for several decades there are still significant efforts required to gain greater understanding of these complex molecules in relation to their structure, bioactivity, and exploitation.

## 2. Results and Discussion

### 2.1. Total Phenolic Content (TPC) and in Vitro Antioxidant Activities

Ethanolic extracts were partitioned exhaustively using HPLC grade water to obtain a hydrophilic fraction, which was freeze dried. This hydrophilic fraction was dissolved in a minimal volume of water and fractionated by molecular weight cut-off (MWCO) dialysis, using a 3.5 kDa membrane. Dialysis was used to fractionate the extracts in order to isolate the low molecular weight regions responsible for activity and also to remove unwanted polysaccharides from the extracts. Phlorotannins were isolated from the low molecular weight (<3.5 kDa) fraction using a reversed-phase (RP) flash chromatography system [36]. The TPC and antioxidant activity of the enriched phlorotannin fractions from each species were tested to demonstrate that activity observed is due to the high abundance of low molecular weight phlorotannins in the extracts. The level of phenolics detected in the flash chromatography-enriched fraction from the four seaweed species (*F. vesiculosus*, *F. serratus*, *H. elongata*, and *C. nodicaulis*) is presented in Table 1. The macroalga with the highest TPC was *F. vesiculosus* (231.95 ± 8.97 μg PGE/mg sample), followed by *F. vesiculosus*, *H. elongata*, *F. serratus* and *C. nodicaulis*; however, there was no significant difference observed between these species. Wang *et al.* [37] also reported high levels of TPC in 70% acetone crude extracts of *F. serratus* (24.0 g PGE/100g) and *F. vesiculosus* (24.4 g PGE/100 g).

**Table 1:** Total phenolic content (TPC) expressed as μg phloroglucinol equivalents (PE), Ferric reducing antioxidant power (FRAP) expressed as μg trolox equivalents (TE), and 1,1-diphenyl-2-picryl-hydrazl (DPPH) radical scavenging ability (RSA) expressed as IC_50_ mg/mL of ethanol/water extracts of four macroalgae samples.

Seaweed Species	TPC (μg PE/mg Sample)	FRAP (μg TE/mg Sample)	DPPH (IC_50_ µg/mL)
*Fucus vesiculosus*	231.95 ± 8.97 ^a^	307.27 ± 1.22 ^a^	4.00 ± 0.01 ^b^
*Himanthalia elongata*	198.28 ± 9.17 ^b^	130.89 ± 0.45 ^b^	14.00 ± 0.04 ^a^
*Cystoseira nodicaulis*	89.14 ± 2.57 ^c^	101.35 ± 0.36 ^b^	28.00 ± 0.01 ^a^
*Fucus serratus*	180.55 ± 16.98 ^b^	110.94 ± 0.65 ^b^	19.00 ± 0.03 ^a^

Values represented as mean ± standard deviation of three assays, performed in triplicate. Different superscript letters (^a,b,c^) indicate a significant difference in Tukey’s HSD test.

Similar trends were observed in the DPPH (2,2-diphenyl-1-picryl-hydrazyl-hydrate) and FRAP (ferric reducing antioxidant power) levels of enriched fractions, with *F. vesiculosus* having the highest FRAP activity (307.27 ± 1.22 μg Trolox Equivalent (TE)/mg sample) and DPPH activity with a subsequent IC_50_ value of 4.00 ± 0.01 µg/mL. *H. elongata* had the next highest FRAP activity (130.89 ± 0.45 μg TE/mg sample) with both *C. nodicaulis* and *F. serratus* having slightly lower levels of 101.35 ± 0.36 μg TE/mg sample and 110.94 ± 0.65 μg TE/mg sample, respectively.

Wang *et al.* also found that *F. vesiculosus* had the highest radical scavenging ability in 70% acetone extracts (EC_50_ = 10.7 × 10^−3^ mg/mL) when compared to 11 other macroalgae species [37]. Overall *F. vesiculosus* had significantly higher (*p* < 0.05) TPC, DPPH, and FRAP activities when compared to the other species investigated. Our studies [38] have also shown that higher molecular weight fractions (3.5–100 kDa) exhibited greater antioxidant and TPC potential than lower molecular weight fractions (3.5 kDa). However, using reversed-phase flash chromatography on the lower molecular weight fraction (<3.5 kDa), a phlorotannin-enriched fraction could be obtained which had increased TPC and antioxidant activity.

### 2.2. UPLC-QQQ-MS Profiling of Phlorotannin-Enriched Fractions

**Figure 1:**
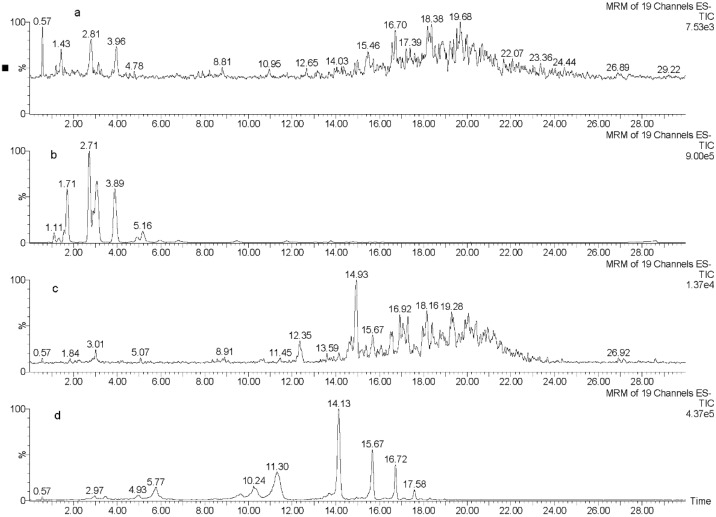
Total ion chromatogram (TIC) of reverse phase (RP) flash chromatography-enriched fractions of (**a**) *Fucus serratus*; (**b**) *Fucus vesiculosus*; (**c**) *Himanthalia elongate*; and (**d**) *Cystoseira nodicaulis*. Prominent peaks are labeled with retention time (on the left or on top) along with observed *m/z* (on the right or below the retention time).

Figure 1 displays a total ion chromatogram (TIC) of reversed-phase (RP) flash chromatography-enriched fractions of the four species under investigation, presenting the structural complexity of the isolated phlorotannins. The characterization of phlorotannins, which in theory could be generated by indiscriminate coupling during biosynthesis, is taxing due to the difficulty in separating complex isomeric mixtures with identical molecular weights and similar structures into individual compounds [37]. Due to the large numbers of isomers present at each level of DP (degree of polymerization) in particular species, it is unlikely that the isolation and subsequent characterization of their structure will be attainable in the near future. Individual structural elucidation of each phlorotannin present in the species investigated in this study would not be possible with current chromatographic techniques. A more feasible aim would be to investigate species containing complex isomeric mixtures by UPLC and mass spectrometric profiling, which has been limited to date [34,39].

As illustrated in Figure 1a, the majority of the phlorotannins present in *F. serratus* had molecular weights between 6–12 phloroglucinol units. Similar to *F. serratus*, the TIC of *H. elongata* (Figure 1c) shows that the majority of the phlorotannins had molecular weights broadly in this range also. The most predominant peak is observed at *m/z* 1117 (DP of 9), with other noticeable peaks being observed at *m/z* 1241 (DP of 10), 1365 (DP of 11), and 1490 (DP of 12). *F. vesiculosus* (Figure 1b) displays a very different profile to that observed for *F. serratus* and *H. elongata*, with the most prominent peaks observed in this enriched fraction at *m/z* 497 (DP of 4), 745 (DP of 6), and 869 (DP of 7)—*i.e.*, at a lower molecular weight range than that of the other two species previously discussed. The profile for *C. nodicaulis* (Figure 1d) is also different to the other species in this study, displaying both a range of very low molecular weight phlorotannin polymers along with higher molecular weight phlorotannins. For example, the most prominent peaks are *m/z* 993 (DP of 8), 1117.40 (DP of 9), and 1241 (DP of 10). It has been reported in the literature that MS analysis has been performed previously on some of the species investigated in this study, *F. vesiculosus* and *C. nodicaulis* [5,33]; however, none have demonstrated successful separation of polymeric phlorotannins with greater than 10 phloroglucinol units. Previous reports have suggested that the molecular weight of phlorotannins influence the antioxidant activity they possess. For example, Hagerman *et al.* [40] reported that high molecular weight condensed (plant) tannins exhibited antioxidant activities 15–30 times greater than simple phenolics and Trolox. They hypothesized that this activity was due to the high molecular weight of these compounds and the proximity of many aromatic rings and hydroxyl groups, which improve the free radical scavenging ability of these compounds. Fan *et al.* [41] also investigated the antioxidative properties of high molecular weight polyphenols from the brown seaweed *Sargassum kjellmanianum*. The results of their study indicated that hydroxyls in phlorotannins are hydrogen donors and the vicinal trihydroxyl is a more active hydrogen donor than meta-trihydroxyl. They thus concluded that the hydroxyl radical scavenging capacity is closely related to the number of sites available in the phlorotannin structure for the addition of hydrogen radicals. Wei *et al.* [42] also investigated the antioxidant activity of high molecular weight phlorotannins from the brown seaweed *Sargassum kjellmanianum*, as well as *Sargassum thunbeergii kuntze*, and found them to show strong scavenging ability, thus proving to be potent marine antioxidants. Samples containing predominantly lower DP exhibited the greatest activity. *F. vesiculosus* contained mostly DP 4–7 and had a significantly greater antioxidant activity than the other three species investigated, composed of DP 9–12. This is likely due to the greater numbers of free hydroxyl groups present, which are not involved in monomer linkages.

While there is little clarity as to the reason behind the different degrees of polymerization exhibited by macroalgae-derived phlorotannins, it may be related to the greater number of resonance stabilization sites present in higher molecular weight compounds, thus giving them stronger antioxidative properties. Therefore the degree of polymerization and the resulting number of isomers could therefore arise from *in situ* production in response to stresses such as UV radiation, herbivore predation, and changes in salinity for intertidal species. However, further investigation would be necessary to confirm this hypothesis.

**Figure 2:**
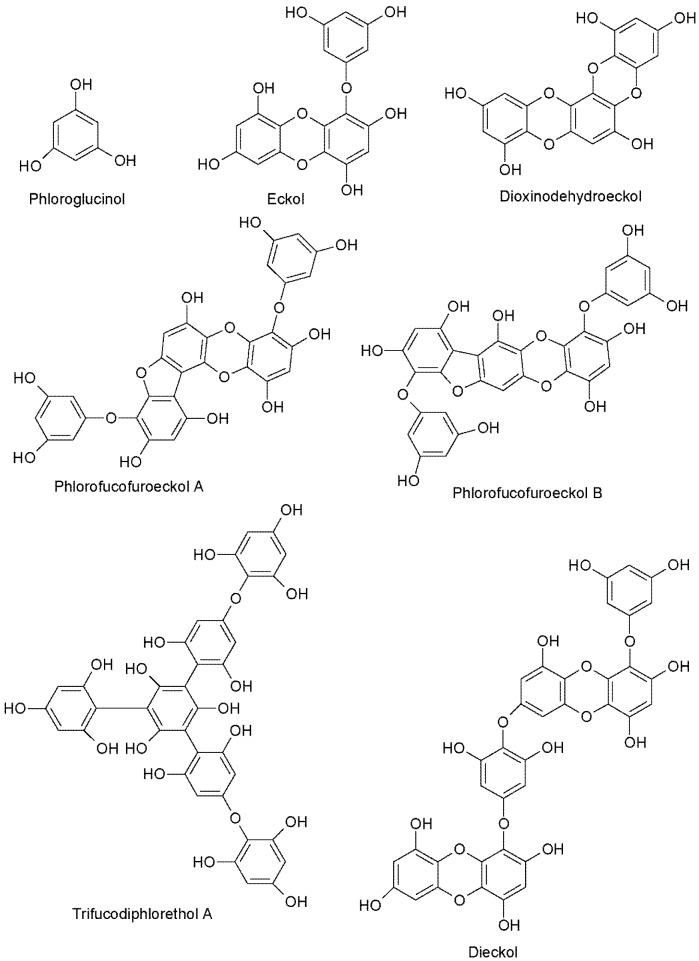
Chemical structures of a selection of phlorotannins, illustrating varying bonding mechanisms between phloroglucinol units.

**Figure 3:**
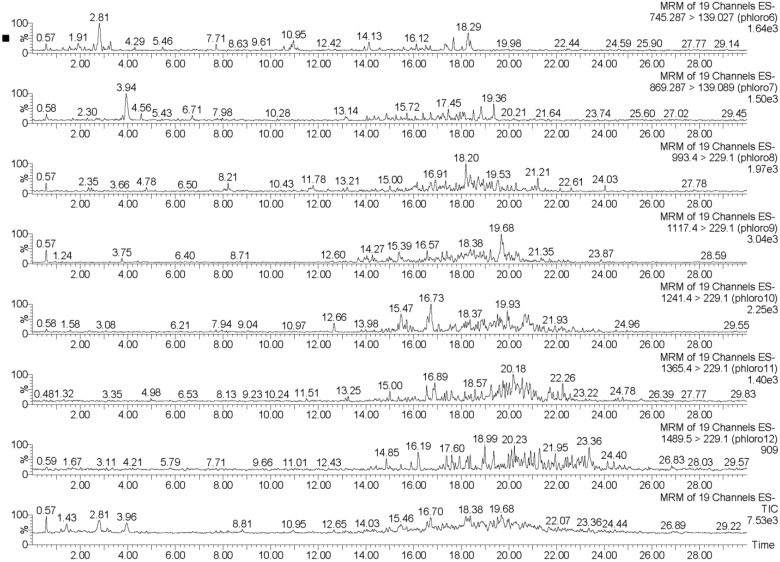
UPLC-MS/MS MRM ion chromatograms of reversed-phase (RP) flash chromatography enriched fraction of *Fucus serratus* for deprotonated molecules [M − H]^−^ of 745.3, 869.3, 993.4, 1117.4, 1242.4, 1365.4, and 1489.4 *m/z*, which correspond to phlorotannins of 6, 7, 8, 9, 10, 11, and 12 phloroglucinol units, respectively.

**Figure 4:**
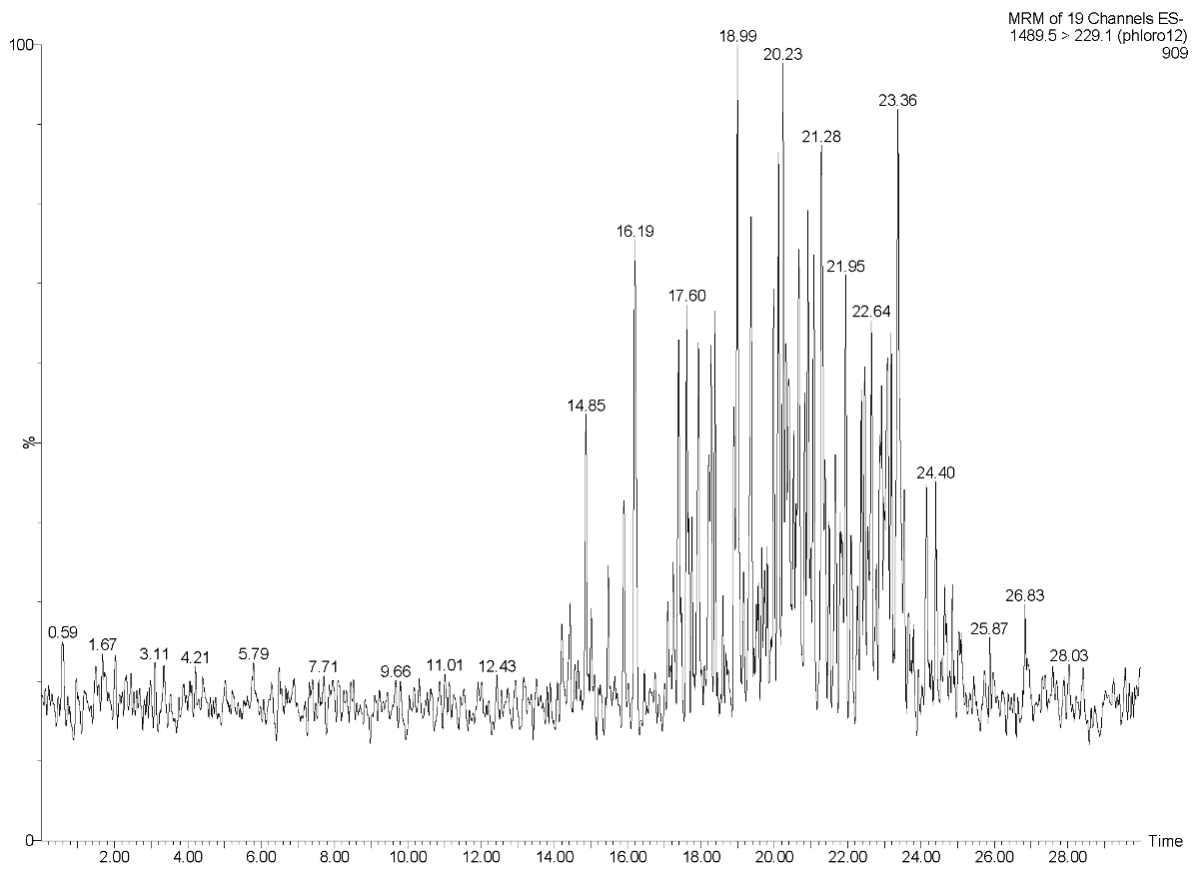
UPLC-MS/MS MRM extracted ion chromatogram of *Fucus serratus* phlorotannin isomers at the selected deprotonated molecules [M − H]^−^ of 1490 *m/z*, which corresponds to phlorotannins consisting of 12 PGUs in size.

The UPLC-QQQ-MS method employed in this study resulted in the detection of phlorotannins in the range of 374–1986 Daltons, using a tandem quadrupole MS in MRM mode for increased sensitivity and for isomer detection. By individually tuning the theoretical molecular weights of phlorotannins while infusing a complex phlorotannin mixture using the IntelliStart™ software, combined with the manual tuning, suitable MRM fragments for each phlorotannin up to 16 phloroglucinol units in negative ionization mode could be determined. It was apparent that the level of phlorotannin isomerization across species differed to some degree. This difference may be due to possible structural features of the phlorotannins present and the species-specific biosynthetic mechanisms. The monomeric units in phlorotannins are linked through aryl–aryl bonds and diaryl bonds, forming different subgroups of phlorotannins [43]. When aromatic rings are connected purely by aryl–aryl bonds, a group of fucols is formed (Figure 2). Phlorethols are formed solely by aryl ether bonds (Figure 2). Fuhalols are constructed by phloroglucinol units that are connected with para- and ortho-arranged ether bridges containing one additional OH-group in every third ring (Figure 2). When there exists at least one three-ring moiety with a dibenzodioxin elements substituted by a phenoxyl group at C-4, the group is named eckols (Figure 2). Carmalols are further derivatives of phlorethols containing a dibenzodioxin moiety. Endofucophlorethols and isofuhalols (Figure 2) are small, distinct, specialized groups. Figure 3 displays the MRM transitions of phlorotannins from *F. serratus*, highlighting the abundance of isomers present for individual deprotonated molecules. A high number of isomers were detected for individual molecular ions in the species *F. serratus*, with 178 isomers in total being detected between DP 6 and DP 14; DP 12 had the highest isomer number of 42. This would suggest a significant variation in branching positions is occurring between the monomeric units. Figure 4 presents a more detailed view of *F. serratus* MRM corresponding to DP of 12 (MS/MS 1489.5–229.1 transition) to show distinctly the complexity of isomerization occurring. Similar to what was observed for *F. serratus*, *H. elongata* has a large number of isomers between DP 7 and DP 12 units; 96 isomers in total were detected in this range, with DP 8 having the highest isomer content of 32. Unlike *F. serratus* and *H. elongata*, *F. vesiculosus* phlorotannins eluted at early retention times and the most prominent peaks were observed in the lower molecular range at DP 4 to DP 9. There were still a significant number of isomers observed in this range, with 144 isomers detected in total. *C. nodicaulis* was similar to *F. vesiculosus* in that the phlorotannins eluted in low percentages of organic solvent. The most prominent peaks are in the range DP 8–11 with a lower number of isomers observed for each deprotonated molecule [M − H]^−^. A total of 48 isomers were detected between the range of DP 4–11. Due to the low level of isomers detected, it may be assumed that there is limited variation in branching between monomeric units.

**Figure 5:**
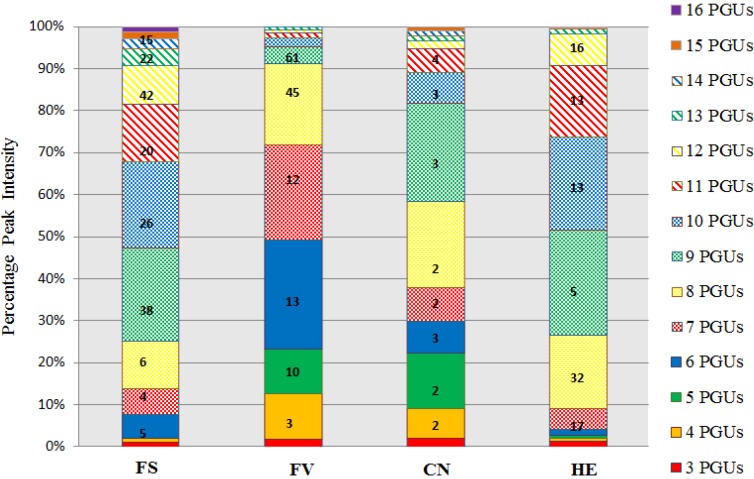
UPLC-MS total percentage peak intensity for individual molecular ions corresponding to phlorotannins of between 3 and 16 phloroglucinol units (PGUs) enriched from four species of macroalgae. The number of phlorotannin isomers detected at each molecular weight correspond to phlorotannins detected fromin extracts from each brown algal species are highlighted with **bold** numbers. *Fucus serratus* (FS), *Fucus vesiculosus* (FV), *Cystoseira nodicaulis* (CN), *Himanthalia elongata* (HE).

The peak intensities and also the number of isomers for each species can be viewed in Figure 5. *F. serratus* and *C. nodicaulis* show similar results, with 90% of the phlorotannins being observed between 3–12 and 3–10 PGUs, respectively. In the species *F. vesiculosus*, the percentage peak intensities observed highlight that a majority of the phlorotannins are of lower molecular weight and up to 90% of the phlorotannins are found to be between 3–8 PGUs. In *H. elongata* up to 80% of the phlorotannins found were between 7–11 PGUs. Previous research to date on the isolation and characterization of individual phlorotannin compounds from some of these species has been limited [31,32,34,37]. This study is the first to emphasize the complex nature of these compounds in relation to the number of isomers present even in these low molecular weight fractions. This outcome also draws attention to the considerable amount of research that would be required for complete characterization of some of the more complex species such as *F. serratus* and *H. elongata*.

**Figure 6:**
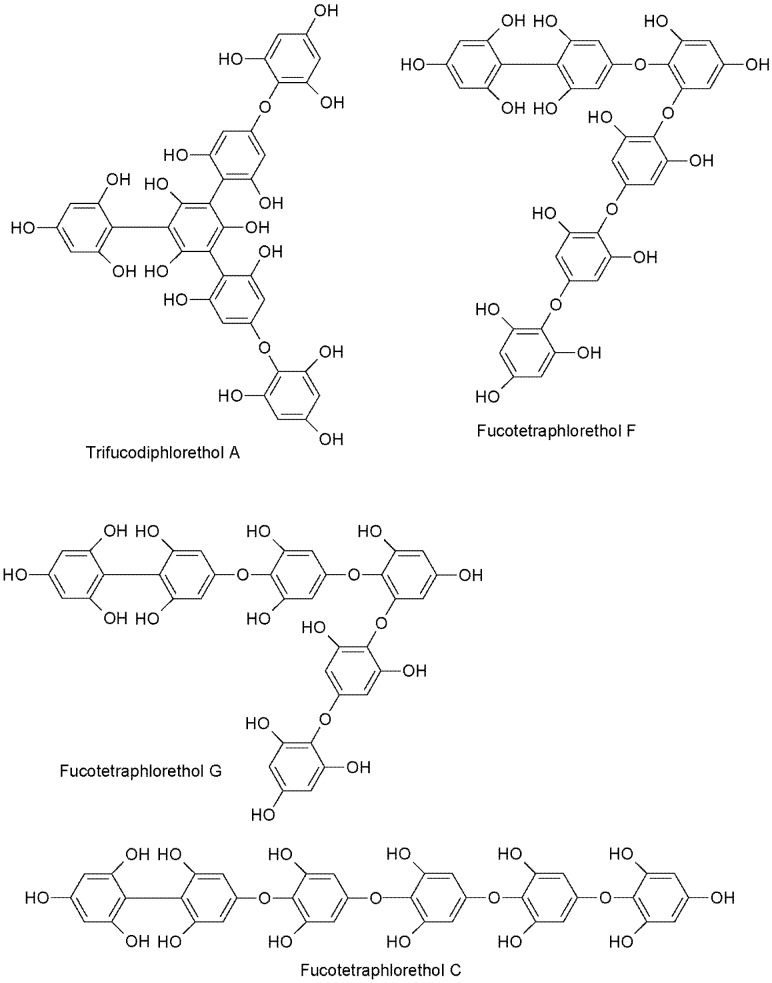
Chemical structure of a range of isomers (fucotetraphlorethol A–G), with degrees of polymerization of 6, demonstrating the potential for large numbers of isomers.

To date only partial analysis of phlorotannin, in a lower molecular weight range in comparison to this study, has been conducted on the species *F. vesiculosus*, *C. nodicaulis*, and *H. elongata*, while no phlorotannins have been characterized from the species *F. serratus*. Ferreres *et al.* [33] used HPLC DAD-ESI-MS to analyze phlorotannins from the species *C. nodicaulis*, with phlorotannin structures ranging from 499–743 *m/z* (4–6 PGUs) detected. Koivikko *et al.* [39] detected a phlorotannin tetramer (497 *m/z* or 4PGUs) in the species *F. vesiculosus* using normal phase separation. Grosse-Damhues *et al.* [44] isolated and characterized an eight-ring phlorotannin from the brown species *H. elongata* using field desorption mass spectrometry. The present study has identified phlorotannins at much higher degrees of polymerization, *i.e.*, up to 16 PGUs, along with observation of the number of isomers for each particular molecular ion, illustrating the complex nature of several of the species investigated. At present only a small number of phlorotannin compounds have been isolated and chemically characterized from these four macroalgae species. In the species *H. elongata* just one compound, difucophlorethol, has been isolated and the structure elucidated at the molecular mass 498.399 (4 PGUs) using a combination of both MS and NMR [43]. Only two compounds, fucotetraphlorethol A [45] (Figure 6) and fucotetraphlorethol B, have been reported at the molecular mass 746.591 (6 PGUs) in the species *H. elongata*. However, for this molecular weight 22 isomers were identified, which would suggest that there are still a substantial number of unreported structures requiring isolation and identification for this particular mass in this species. At the molecular mass 622.49 (6 PGUs), just one compound, fucotriphloroethol C [45], has been reported in the species *H. elongata*. In the species *F. vesiculosus*, a greater number of phlorotannin compounds have been reported. For example, Pary *et al.* [46] isolated two phloroglucinol derivatives belonging to the class of fucophlorethols, along with the previously known fucotriphlorethol A, from the ethanolic extracts of the brown macroalgae *F. vesiculosus* and investigated their chemopreventive potential. Two compounds, fucodiphlorethol A and fucodiphlorethol E [47] have been isolated and structurally elucidated using MS and NMR with the molecular mass 498.399 (4 PGUs) and five compounds have been characterized at the molecular mass 746.591 (6 PGUs), fucotetraphlorethol C, D, E, F and G [48]. This study has identified 13 isomers for this particular molecular weight range, demonstrating the limited amount of structural elucidation that has occurred for these macroalgal species. To date limited research has been carried out on the identification of phlorotannin compounds from the species *F. serratus* without details on the level of isomers, while studies on *C. nodicaulis* have not been reported. This is most probably due to the complex nature of these species and also the level of isomerization observed. Previous reports on phlorotannin compounds have merely suggested that they are responsible for observed activities [5,7,15,19]. Previous work done by this group [38] generated tannin-enriched fractions using reversed-phase flash chromatography and LC-QTOF-MS identified a high abundance of low molecular weight phlorotannins in the <3.5 kDa fractions from the species *F. serratus*. The only reports of work on the species *C. nodicaulis* was carried out by Ferreres *et al.* [33], where eight compounds in the *m/z* range of 497–743 (fucophlorethol, tridiphloroethol, fucotriphloroethol, 7-phloroekol, phlorofucofuroeckol, and bieckol/dieckol) were detected in a range of brown algae species including *C. nodicaulis*. They observed similar results to the present report: three isomers were detected for the molecular ion 499 (4 PGUs), compared to two isomers in this study. However, this study detected phlorotannin compounds in this species between the range of 3–16 PGUs, thus providing full profiling of the low molecular weight phlorotannins present in this species.

## 3. Experimental Section

### 3.1. Standards and Reagents

All chemicals used were reagent grade; 2,2-diphenyl-1-picrylhydrazyl (DPPH), gallic acid, ferrous chloride, Ferrozine, 2,4,6-tris(2-pyridyl)-*s*-triazine (TPTZ), 6-hydroxy-2,5,7,8-tetramethylchroman-2-carboxylic acid (Trolox), and glass wool were obtained from Sigma-Aldrich Chemical Co. (Arklow, Wicklow, Ireland). All solvents used were HPLC grade. BioDesignDialysis Tubing™ with 3.5 kDa cut-off was acquired from Fisher Scientific (Dublin, Ireland). Agilent SuperFlash™ SF2555G C18, 50 µm were obtained from Apex Scientific (Maynooth, Ireland).

### 3.2. Samples

The brown macroalgae samples used in this study were identified by a trained phycologist, Dr. Soler-Vila (NUI Galway), and harvested off the west coast of Ireland. *Fucus serratus* (Fucaceae) was harvested from Finnavarra, Clare, Ireland in the summer of 2011, *Fucus vesiculosus* (Fucaceae) was harvested from Spiddal, Galway, Ireland in the autumn of 2010, *Himanthalia elongata* (Himanthaliaceae) was harvested from Finnavarra, Clare, Ireland in spring 2010, and *Cystoseira nodicaulis* (Fucaceae) was harvested from Finnavarra in the summer of 2012. A random selection of a large number of different plants were taken from the shore, to allow for natural variability; these were packed in cool boxes and transported immediately to the laboratory. Samples were washed thoroughly with fresh water to remove sand and epiphytes and were then stored in the freezer at −20 °C. A freeze-dried sample of each was retained for reference at the Irish Seaweed Centre at National University of Ireland, Galway. The macroalgal samples were subsequently freeze-dried, ground to a powder using a Waring^®^ blender (New Hartford, CT, USA), and stored in vacuum-packed bags at −80 °C prior to extraction.

### 3.3. Solid-Liquid Extraction (SLE)

Solid-liquid extraction was employed to extract the phlorotannins from the macroalgae under investigation using ethanol/water (80:20), as this solvent system has previously been shown to be effective for extracting phlorotannin compounds from macroalgae [6,38,49]. Crude extracts were prepared by placing 150 g of the seaweed powder in a conical flask and adding the extraction solvent at a ratio of 10:1 (v/w). The mixture was then placed into a shaker (Thermo Scientific MaxQ6000) at 150 rpm and room temperature for 24 h. Extracts were filtered three times over a 24 h period through a large glass funnel packed with glass wool (Sigma-Aldrich), and after each filtration the residue was replenished with fresh solvent. The extracts were pooled and ethanol was removed using a large scale rotary evaporator (Büchi Rotavapor R-200 with a V 710 vacuum pump, Flawil, Switzerland) with the water bath set at 50 °C. Phlorotannins have been shown to be thermally stable at this temperature [50]. The remaining aqueous portions of the extracts were frozen and freeze-dried. All extracts were subsequently ground to a fine powder using a mortar and pestle prior to use.

### 3.4. Partitioning and Molecular Weight Cut-off (MWCO) Dialysis

A hydrophilic fraction of the freeze-dried powder was prepared by exhaustively extracting the powder with HPLC-grade water (20 mL). Periodically, the water was decanted and replaced with fresh water. This was repeated until no further material was extracted leaving behind an oily, highly pigmented residue (hydrophobic fraction). The water washes were pooled and freeze-dried to give a hydrophilic fraction.

The hydrophilic fraction was further fractionated using MWCO dialysis (BioDesignDialysis Tubing™) into dialysates containing compounds with MWs <3.5 KDa and >3.5 KDa. To do this the hydrophilic (water-soluble) fraction was dissolved in a minimal volume of deionized water and decanted into 3.5 kDa dialysis tubing clamped at one end. The tubing was clamped at the other end, immersed in a reservoir of deionized water, and shaken moderately (50 rpm) at room temperature for 72 h. The reservoir of water was refreshed periodically until no further color was visible in the dialysate. Both the high molecular weight (>3.5 kDa) and low molecular weight (<3.5 kDa) dialysates were then frozen and freeze-dried. The <3.5 kDa fraction was further purified using reversed-phase flash chromatography; the >3.5 kDa fraction was stored and subjected to *in vitro* antioxidant testing.

### 3.5. Reversed-Phase Flash Chromatography

The lower molecular weight (<3.5 kDa) fraction of each species was further fractionated using a two-step reverse phase (RP) flash chromatography method, as described elsewhere [36]. RP-flash chromatography was carried out on an Analogix Intelliflash 310 system (Agilent Technologies, Dublin, Ireland) using a Agilent SuperFlash™ SF25-55G C18 with a sorbent mass of 55 g and a mean particle size of 40–60 μm. One gram of the <3.5 kDa fraction material was dissolved in water and loaded onto the column. A two-step elution gradient was employed. The mobile phase consisted of the primary eluent of HPLC grade water (0–20 min) and the secondary eluent of 100% methanol (20–40 min). The flow rate was 50 mL/min. Elutant was collected from 0–20 min (fraction 1) and from 20–40 min (fraction 2). UV detection was observed at 210, 225, and 250 nm. A phlorotannin polymer fraction was collected from 20 to 40 min.

### 3.6. Total Phenolic Content (TPC)

The TPC of fraction 2 was quantified according to the method of Singleton [51]. Enriched fractions were diluted in methanol and tested at 1 mg/mL. Briefly, a 100 μL aliquot of sample was mixed with 100 μL Folin–Ciocalteu phenol reagent, 100 μL methanol, and 700 μL 20% Na_2_CO_3_. The reaction mixture was mixed thoroughly and allowed to stand for 20 min at room temperature in the dark. Samples were centrifuged at 13,000 rpm for 3 min and the absorbance of all samples was measured at 735 nm using a Hitachi U-2900 Spectrophotometer. Standard solutions of gallic acid in methanol ranging from 10–200 μg mL^−1^ were used to construct a standard curve. TPC was expressed in terms of microgram gallic acid equivalents per milligram of dry weight sample (μg GAE mg^−1^ sample). All fractions were tested in triplicate.

### 3.7. In Vitro Antioxidant Activity

#### 3.7.1. Ferric Reducing Antioxidant Power (FRAP)

The FRAP of the low molecular weight phlorotannin fractions was assessed according to a previously described method with slight modifications [52]. This method allows determination of the ferric reducing ability (µmol Fe^(III)^ converted into Fe^(II)^) in aqueous solutions of the samples, as a measurement of their antioxidant power. The FRAP reagent contained 10 mL of 10 mmol/L TPTZ (2,4,6-tri(2-pyridyl-5-triazine) solution in 40 mmol/L HCl plus 10 mL of 20 mmol/L FeCl_3_·6H_2_O and 100 mL of 0.3 mol/L acetate buffer, pH 3.6. A 2 mM trolox stock solution was prepared and diluted with methanol to give concentrations ranging from 0.1–0.4 mM. In brief, 180 μL of freshly prepared FRAP reagent at 37 °C was pipetted into a 96-well microtiter plate containing either 20 μL test sample or standard (or methanol for the blank). Samples were tested at 1 mg/mL. Samples were incubated at 37 °C for 40 min and then the absorbance was measured at 595 nm using a plate reader (BMG Labtech FLUOstar Omega microplate reader system, GmbH, Ortenberg, Germany). Trolox was used as standard and FRAP values were expressed as microgram trolox equivalents per milligram dry weight sample (μg TE/mg dry weight sample).

#### 3.7.2. DPPH (1,1-diphenyl-2-picryl-hydrazyl) Scavenging Activity

The free-radical scavenging capacity of the phlorotannin fractions was analyzed using the DPPH assay according to Goupy [53]. Two hundred microliters of each sample were pipetted into a well of the plate and serial dilutions of the phlorotannin samples starting at 2 mg/mL in well 1 were prepared in the subsequent wells. One hundred micro liters of a 1 in 5 dilution of the DPPH/methanol (0.238 mg of DPPH per milliliter of methanol prepared daily) working solution was pipetted into each well. The plate was then placed in a dark place at room temperature for 30 min. The absorbance was measured at 515 nm using a plate reader (BMG Labtech FLUOstar Omega). The decrease in absorbance of the sample was calculated by comparison to a control (100 μL sample extraction solvent and 100 μL DPPH). The relative decrease in absorbance (PI) was calculated using equation 1 below: trolox at concentrations ranging from 0.1–0.4 mM was used to construct a standard curve, to determine the IC_50_ value of trolox, and to ensure methods and solutions were correct.

*PI (%)* = *[1 − (Ae/Ab)]* × *100* (1)

where Ae means absorbance of sample extract and Ab means absorbance of control. PIs were used to calculate the relative antioxidant activity according to the method to Ollanketo [54]—*i.e.*, where PI1 (superior) and PI2 (inferior) were used to estimate the concentration of extract required to result in a 50% decrease of DPPH· bsorbance. Antioxidant activity was expressed as Antiradical Power (ARP), which is the reciprocal of the IC_50_ (mg mL^−1^) used to define the concentration of sample extract that produces a 50% reduction of the DPPH radical absorbance [55]. High ARP values indicate the strong radical scavenging ability (RSA) of a sample [55].

### 3.8. Statistical Analysis

All phlorotannin fractions were analyzed in triplicate. Measurement values are presented as means ± standard deviation. One-way analysis of variance (ANOVA), followed by the Tukey *post hoc* comparison test, was carried out to test for significant differences in antioxidant activity and phenolic content between macroalgal species using the statistical program Minitab^®^ Release 15 for Windows.

A probability value of *p* < 0.05 was considered statistically significant.

### 3.9. UPLC-ESI-MS of Phlorotannin-Enriched Fractions

UPLC-MS analysis of *F. serratus*, *F. vesiculosus*, *C. nodicaulis*, and *H. elongata* ethanol/water phlorotannin-enriched fractions (prepared as described in sections 3.2–3.5) was undertaken using an Acquity™ UPLC^®^ System (Waters Corporation, Micromass MS Technologies, Manchester, UK). The system comprised of a binary pump solvent manager with the ability to generate pressures up to 15,000 psi, coupled with an Acquity™ TQD-MS (Waters, Dublin, Ireland) It was run in electrospray negative ion mode with multiple reactions monitoring (MRM), which was developed and optimized with a phlorotannin-enriched sample using the IntelliStart™ software (Waters, Dublin, Ireland) according to molecular masses of phlorotannins containing 3–16 (maximum permitted with TQD) units of phloroglucinol. For separation a Waters Aquity™ HSS PFP column (100 Å, 1.8 μm particle size, 2.1 mm × 100 mm) was used and maintained at 40 °C. The mobile phase consisted of 0.1% formic acid in an aqueous solution (A) and acetonitrile with 0.1% formic acid (B). The flow rate was set at 0.5 mL/min and the injection volume was 5 μL. Elution was performed as follows: 0.5% B from 0 to 10 min, 0.5%–30% B from 10 to 26 min, 30%–90% B from 26 to 28 min, and 0.5% B from 28 to 30 min. The parameters from the MS were as follows: capillary voltage, 2.8 kV; source temperature, 350 °C; desolvation temperature, 50 °C; desolvation gas, 800 L/h [33]. Conditions for multiple reaction monitoring (MRM) as determined by a combination of IntelliStart™ and manual tuning are listed in Table 2.

**Table 2:** MRM conditions for TQD-MS analysis of phlorotannins containing 3–16 units of phloroglucinol, determined by a combination of IntelliStart™ and manual tuning.

PGU’s	Precursor Ion (*m/z*)	Product Ion (*m/z*)	Cone Voltage (V)	Collision Voltage (eV)
3	373.1	139	40	34
4	497.2	139	40	40
5	621.2	139	42	42
6	745.3	139	56	40
7	869.3	139	56	52
8	993.4	229.1	60	50
9	1117.40	229.1	70	50
10	1241.40	229.1	70	52
11	1365.40	229.1	70	52
12	1489.50	229.1	70	54
13	1614.00	229.1	70	54
14	1738.30	229.1	70	56
15	1862.30	229.1	70	56
16	1986.40	229.1	70	56

## 4. Conclusions

This present study profiled the phlorotannin metabolite composition in macroalgae-derived phlorotannins and determined the degree of isomerization of compounds between 3–16 phloroglucinol monomers in four species (*F. serratus*, *F. vesiculosus*, *H. elongata*, and *C. nodicaulis*). In general, a significantly higher number of isomers were detected corresponding to phlorotannins of much higher degree of polymerization (up to 16 monomer units) in comparison to previous reports. The majority of the LMW phlorotannins found in *F. serratus*, *F. vesiculosus*, *C. nodicaulis*, and *H. elongata* had molecular weight ranges from 6–12, 4–8, 7–12, and 4–11 monomers, respectively. Purification of extracts using MWCO dialysis and reversed-phase flash chromatography for the removal of carbohydrates, combined with the use of a PFP column during UPLC-MS analysis, allowed for distinctive metabolite profiles to be elucidated and polymeric phlorotannin isomers to be detected. This allowed for a more complete picture of the phlorotannin composition of individual macroalgae species. The work has demonstrated that even in relatively LMW fractions, these molecules are highly complex; this complexity poses a real challenge in terms of further purification and chemical characterization of individual compounds.

Separation of longer condensed tannins is a difficult task and is complicated by the increase in the number of isomers with increasing polymer length. At present, there are no reports suggesting branching may influence biological activity of phlorotannins. However, the results presented here have suggested that due to the complex level of isomerization demonstrated within these species, investigation to link biological activity to a particular isomer may be a difficult task. In addition, the biological rationale behind this structural complexity has yet to be elucidated, if there is one. As well as molecular weight, seasonal variation may also affect the profile and biological activity of phlorotannin profiles. However, variations in phlorotannin concentrations among the development stages of brown algae have been reported [47]. Further study may also highlight particular environmental factors that may affect the phlorotannin metabolite profile in individual macroalgal species. The present study has highlighted that UPLC-MS/MS can serve as a valuable tool for investigating factors that influence the range of molecular weights and isomeric complexity present in macroalgae-derived phlorotannins.

This might be due to the fact that UPLC was utilized, allowing increased efficiency in separation facilitating the profiling of phlorotannin isomer for oligomers between DP of 3–16 phloroglucinol units.
